# Palliative radiation therapy in patients with metastasized pancreatic cancer - description of a rare patient group

**DOI:** 10.1186/2047-783X-19-24

**Published:** 2014-05-13

**Authors:** Daniel Habermehl, Ingo C Brecht, Jürgen Debus, Stephanie E Combs

**Affiliations:** 1 Department of Radiation Oncology, Klinikum rechts der Isar, TU München, Ismaninger Strasse 22, 81675 Munich, Germany; 2 Department of Diagnostic Radiology, University Hospital of Heidelberg, Im Neuenheimer Feld 110, 69120 Heidelberg, Germany; 3 Department of Radiation Oncology, University Hospital of Heidelberg, Im Neuenheimer Feld 400, 69120 Heidelberg, Germany

**Keywords:** Pancreatic cancer, Radiotherapy, Palliation, Palliative radiotherapy, Oligometastasis

## Abstract

**Background:**

Pancreatic cancer (PAC) patients experience a high rate of locoregional recurrences and distant metastasis finally leading to their demise even after curatively-intended multidisciplinary treatment approaches including surgery, chemotherapy and radiotherapy. However, clinical reports on bone and brain metastases in PAC patients are extremely rare and thus timing and dose description are not well defined. Our work therefore summarizes a mono-institutional experience on the use of radiotherapy (RT) for PAC patients with metastatic disease with the aim of identifying overall survival and treatment response in this rarely reported patient group.

**Method:**

Forty-four PAC patients with 66 metastatic lesions were treated with palliative radiotherapy (RT). Thirty-three patients (48 lesions), 7 patients (11 lesions) and 5 patients (7 lesions) with bone, liver and brain metastases analyzed respectively were analyzed; one patient had both bone and cerebral metastases and was treated for the lesions, thus including him in both subgroups. Indications for RT were pain, neurological impairment, risk of pathological fracture or imminent danger for development of any of these conditions in case of tumor progression. Median age was 64 years (range 38 to 78 years) and there were 27 male (61%) and 17 (39%) female patients. Analyses of overall survival (OS) and local control were performed. OS was calculated from the first day of RT.

**Results:**

Median overall survival (mOS) of all patients after start of RT was 4.2 months. Survival rates after 1, 3 and 6 months were 79.3%, 55.3% and 30.3% respectively. Patients presenting with bone metastasis had a mOS of 3.1 months and after 1, 3 and 6 months, survival rates were 75.3%, 46.5% and 19.9% respectively. Symptomatic response to therapy was recorded in 85% of all evaluated patients with bone metastasis. Patients undergoing radiosurgery because of liver metastasis were locally controlled in all but one patient after a median follow-up of 8.3 months.

**Conclusion:**

Overall survival of all patients with metastatic disease was considerably worse. A major goal for the future must be the selection of an appropriate RT treatment in terms of duration and technique for these PAC patients.

## Background

Pancreatic cancer (PAC) patients experience a high rate of locoregional recurrences and distant metastasis finally leading to their death even after curatively-intended multidisciplinary treatment approaches including surgery, chemotherapy and radiotherapy [1,2]. To date, concepts with pure palliative aim can be separated from semi-curative treatment for oligometastasized lesions due to the biological differences of the underlying disease. Local intensified treatment options of isolated local recurrences consist of either radical surgical approaches or neoadjuvant chemoradiation in case of unresectability [3-5]. In cases of peritoneal metastasis which frequently occur or in cases of distant metastasis predominantly to the liver and lungs, systemic therapy with, for example, FOLFIRINOX or gemcitabine (GEM) or other novel approaches including molecular targeted treatments, is the palliative treatment of choice with impact on overall survival (OS) compared to observation alone [6]. The landmark trial of the PRODIGE-group that introduced FOLFIRINOX as the new standard of care for PAC patients with metastatic disease showed a median overall survival (mOS) of approximately 11 months compared to 6.8 months for the GEM group; however only patients with a good performance status (ECOG 0 or 1) were included. This explains the worse outcome with a mOS of only 5.7 months in the GEM-group of the initial trial of [7] that favored GEM over fluorouracil [7]. As clinical trial patient groups are highly selected, one may consider a shorter OS for many patients after diagnosis [8,9].

Whereas liver, lung and peritoneal metastases are often diagnosed in PAC patients during the treatment or follow-up period, knowledge on the incidence of bone, soft tissue and brain metastases is limited. While brain metastases can lead to life threatening conditions by inducing perifocal edema and to a sudden decline in vigilance or headaches, seizures, nausea and dizziness, bone metastases may cause pain and can lead to pathological fractures and spinal cord compression with consecutive serious neurological symptoms [10,11]. However, palliative radiotherapy has shown high efficacy in palliation and prevention of symptoms in gastrointestinal cancer [12,13].

Today, clinical reports on bone and brain metastases in PAC patients are relatively rare and thus timing and dose description regimens in this tumor entity are not well defined [14-16]. Especially in the palliative setting, intensity and appropriateness of any therapy has to be well chosen and adapted to the patients’ individual prognosis to avoid overtreatment by applying longer treatment schedules than are necessary [17,18].

To provide a data basis for subsequent clinical study concepts in the oligometastasized situation as well as patients with a disseminated disease status, our work summarizes a mono-institutional experience on the use of radiotherapy (RT) for PAC patients with metastatic disease with the aim of determining survival after RT and treatment response in this rarely reported patient group.

## Methods

### Patient characteristics

From 1997 to 2011, a total of 44 PAC patients with metastatic disease incorporating 66 lesions were treated with palliative radiation therapy (RT). Hereof, 33 patients (48 lesions) presented with bone metastases, 7 patients (11 lesions) with liver metastases, and 5 patients (7 lesions) with brain metastases. Indications for RT depended on the location of the lesions and included pain, neurological impairment, risk of pathological fracture or imminent danger for development of any of these conditions in case of tumor progression as a means of ‘prospective palliation’. All patients were discussed in an interdisciplinary setting. Median age was 64 years (range 38 to 78 years) and there were 27 male (61%) and 17 (39%) female patients. All patient characteristics are shown in Table 1. Our study was approved by the Institutional Review Board/the independent Ethics Committee of the Medical Faculty Heidelberg (reference number: S-483/2011).

**Table 1:** Patients with bone metastasis - details and treatment characteristics

**Lesion and treatment characteristics**	**Number**
Radiotherapy indication	
Patient number	33
Lesion number	48
Pain	44/48 (92%)
Instability	24/48 (50%)
Neurological impairment	4/48 (8%)
Radiotherapy	
Overall dose (median, range)	30 Gy (3 to 40 Gy)
Single dose (median, range)	3 Gy (2 to 8 Gy)
Number of fractions (median, range)	10 (1 to 20)
Number of applied doses ≥ 30 Gy	38
Number of treatment schedules ≥ 20 days	10
Most frequent treatment schedules	10 x 3 Gy, 26 lesions (54%)
Short Protocol (SF 4 or 8 Gy)	5/48 lesions (10%)
Conventional fractionation (SF 2/2.5 Gy)	11/48 lesions (23%)
Treatment duration (median, range)	15 (1 to 30 days)
Early discontinuation	7/48 lesions (15%)
Previous systemic treatment	26 (79%)
Symptom response	18/33 (55%)
Localization	
Spine	30 (63%)
Upper/lower extremities	2 (4%)
Pelvis	8 (17%)
Skull base	3 (6%)
Ribs	2 (4%)
Shoulder	4 (8%)
Spine/sacrum	1 (2%)
Spine/ribs	1 (2%)

### Radiotherapy

Treatment of bone, brain and liver metastases was performed using LINAC-based external-beam radiotherapy. For brain metastases, fixation with an individual mask fixation was performed as described previously [2,19]. For whole brain radiotherapy (WBRT), two opposed lateral fields were applied. Stereotactic radiosurgery (SRS) was performed after individual mask fixation using Scotchcast material, and computed tomography (CT) imaging was performed with the stereotactic base frame attached to the mask [19,20]. CT imaging with and without contrast enhancement, as well as contrast-enhanced magnetic resonance imaging (MRI) were used for target volume delineation. We defined the gross tumor volume (GTV) as the contrast-enhancing lesion on CT and MRI, and added a planning target volume (PTV) of 1 to 2 mm. Dose prescription was performed according to the size and the location of the lesion following the guidelines by Shaw *et al*. [21].

Confirmation of bone metastasis was performed radiologically by CT and in some cases also by bone scintigraphy.

As described above, the standard radiotherapy protocols include fractionation regimen of 10 × 3 Gy (20 lesions, 30%) and 20 x 2 Gy (18 lesions, 27%) (Table 2). In 49% of all treatments (33/67 lesions), a hypofractionation protocol was applied with single fractions between 2.5 and 4 Gy. In 42% of the irradiated metastases (28/67 lesions), a normofractionated schedule with single fractions of 1.8 Gy or 2 Gy were applied. In a further six cases, patients firstly underwent a normofractionation but then switched to a hypofractionated protocol because of a worsening of the performance status.

**Table 2:** Patients with liver metastasis - details and treatment characteristics

**Patient and treatment details**	**Number**
Patient number	7
Number of lesions	11
Age (median, range) (years)	64 (53 to 78)
Irradiation of two lesions in one session (patient number)	4
Metastases outside liver (patient number)	2
Radiotherapy	
Stereotactic radiosurgery (number of patients/lesions)	6/9
Tomotherapy (number of patients/lesions)	½
Dose at 80%-isodose (median, range) (Gy)	24 (20 to 28)
RS dose	Number of lesions
20 Gy	2
22 Gy	1
24 Gy	5
26 Gy	1
28 Gy	2
Previous systemic treatment	5 (71%)

RS, radiosurgery.

Radiotherapy was usually performed five days per week. The median treatment time was 15 days. In case of radiotherapy to bone metastases in the spine, the target volume included the non-invaded adjacent vertebral bodies (cranial and caudal direction).

A total of 35 patients were previously treated with systemic agents (predominantly gemcitabine-containing regimens) and none of them received concomitant systemic treatment during palliative RT according to our institutional guidelines.

Liver metastases were irradiated using our in-house standard protocol as described previously [4,5,22]. In brief, patients were immobilized using an individually shaped vacuum pillow and an abdominal compression to reduce the liver movement. A contrast agent enhanced CT scan and a 4D-CT series for quantifying liver motion was acquired for treatment planning. The extracranial stereotactic set-up has been developed at the German cancer research center (dkfz) and is commercially available (Leibinger, Freiburg, Germany). The patient is positioned in an individually shaped vacuum pillow (Brandis Medizintechnik, Weinheim, Germany). The intra-corporal movement of the liver was reduced by epigastric compression using a triangular Plexiglas or carbon plate. Fixation of the plate is performed by two bars, which are firmly attached to the metal or carbon arch. A Siemens Somatom Plus 4 (Siemens, Erlangen, Germany) was used for treatment planning. A spiral CT scan with 5-mm slice thickness and 500-mm field of view was performed which included the localization system. The patients were advised to breathe normally during the scanning time without taking deep breaths.

On the treatment day, patients were repositioned in the above mentioned setting using pen marks and another control CT scan. Until the year 2003, CT imaging was performed offline, and, if positioning was adequate; patients were brought to the linear accelerator (LINAC) using an individual shuttle system leaving the patient in the vacuum bag/abdominal press fixation. In recent years (2004 to 2009), LINACs (for example, Siemens Artiste, Siemens Healthcare, Erlangen, Germany) were equipped with on-board imaging as, for example, in case of tomotherapy, a combination of the 6 MeV LINAC with CT imaging. Target and OAR contouring was performed using Siemens Dosimetrist and Oncologist software (Siemens Sector Healthcare, Erlangen, Germany), and inverse treatment planning was conducted applying the Hi-ART Tomotherapy planning software (TomoTherapy Inc., Madison, WI, USA). Dose constraints for adjacent organs at risk and the liver were used according to Emami *et al*. and Dawson *et al*. [23,24]. In the current analysis we included data on patients which were already described in a previous report on radiosurgery (RS) for liver metastasis from our institution [4,5].

### Follow-up and statistics

Overall survival was calculated from the first day of irradiation until death. Information on follow-up with radiological data on treatment response was available in 29 of 44 patients (66%). The log-rank test was implemented to compare survival curves evaluating the association between clinical variables of interest and survival. All calculations were performed using the statistical software program SPSS 18.0 for Windows (Chicago, IL, US).

## Results

**Figure 1:**
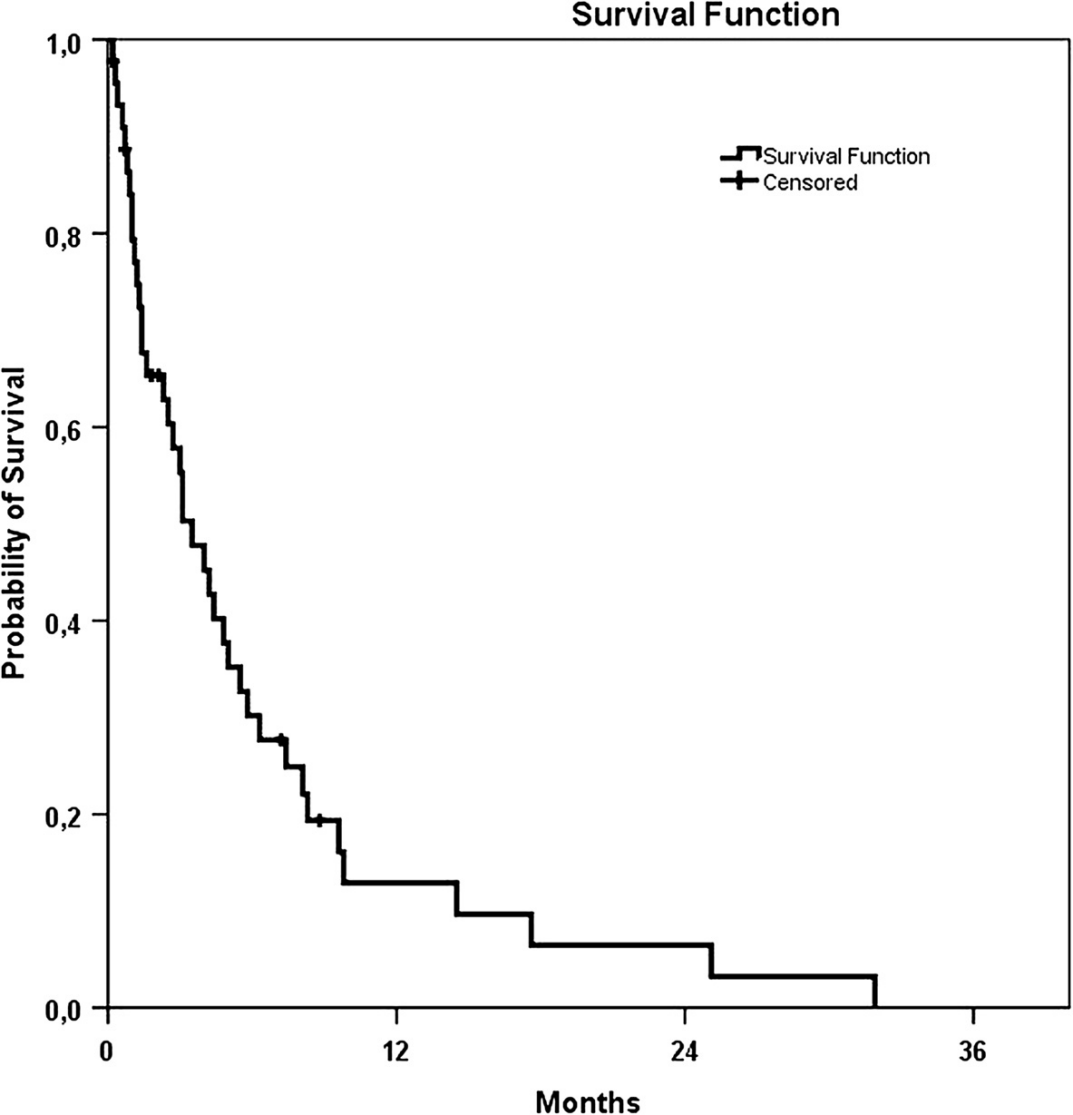
Kaplan-Meier survival curve of all patients.

Median survival of all patients was 4.2 months (95% confidence interval (CI) 2.4 to 6.0). Survival rate after 1, 3 and 6 months was 79.3%, 55.3% and 30.3% respectively (Figure 1).

### Patients with bone metastasis

There was a total number of 33 patients with 48 bone lesions. Lesion and treatment details are listed in Table 1. Patients presenting with bone metastasis had a mOS of 3.1 months (95% CI 1.9 to 4.3) and after 1, 3 and 6 months, survival rates were 75.3%, 46.5% and 19.9% respectively. RT indications were as follows: pain in 44 of 48 lesions (92%), spinal instability in 24 of 48 cases (50%) and consecutive neurological impairment in 4 cases (8%). Localization of the bone lesions was predominantly the spine in 30 cases (63%), the pelvis in 8 cases (17%) and the upper and lower extremities in 2 (4%), the shoulder in 4 (8%), ribs in 2 (4%) and the skull base in 3 (6%) cases. Treatment protocols varied but most patients were treated with 30 Gy in 10 fractions (26 lesions, 54%). Treatment protocols with single doses of 4 and 8 Gy were documented in 5 lesions (10%), while conventional fractionation with single doses of 2 or 2.5 Gy were applied in 11 cases (23%). The median number of applied fractions was 10 (range 1 to 20). In 38 cases (86%) the prescribed overall dose was greater than or equal to 30 Gy. Median treatment duration was 15 days (range 1 to 30 days). An early discontinuation was recorded for 8 treatments (17%). Symptomatic response to RT was confirmed in at least 26 of 30 cases (87%). There was no clinical follow-up for the remaining patients.

### Patients with brain metastasis

A total of five patients with seven brain metastases were analyzed. The median age was 59 years (range 41 to 77) and two patients had multiple lesions (defined as more than 3 lesions synchronously) (Table 3). Three patients had one to three metastases at the time of RT. In all patients there were additional confirmed extra-CNS metastases. Mean survival of patients was 6.3 months (95% CI 3.9 to 8.6). A total of two patients with four cerebral lesions underwent LINAC-based stereotactic radiosurgery with doses of 18 Gy and 20 Gy prescribed to the surrounding 80%-isodose line. Localization of these four lesions was the cerebellum, the right and left parietal lobe and the left frontal lobe. WBRT, because of multiple intracerebral lesions was performed in three patients with a total dose of 30 Gy in 10 fractions (two patients) or 40 Gy in 20 fractions applied with two lateral opposed beams. Three out of four evaluated patients responded to RT in terms of improved neurologic function or reduced lesion size. One patient had a progressive intracerebral lesion at time of the first follow-up MRI.

**Table 3:** Patients with brain metastasis - details and treatment characteristics

**Patient and treatment details**	**Number**
Patient number	5
Number of lesions	7
Age (median, range) (years)	59 (41 to 77)
Multiple lesions (≥3 lesions; patient number)	2
One to three lesions (patient number)	3
Extra-CNS metastases (patient number)	5
Radiotherapy	
Stereotactic radiosurgery (number of patients/lesions)	2/4
Dose at 80%-isodose (median, range) (Gy)	20 (18 to 20)
WBRT (number of patients)	3
Previous systemic treatment	4 (80%)

WBRT, whole brain radiotherapy.

### Patients with liver metastasis

Seven patients with eleven lesions were treated with high-dose stereotactic radiosurgery (six patients with nine lesions) or helical intensity-modulated radiosurgery (tomotherapy, one patient with two lesions) (Table 2). The median prescribed dose to the 80%-isodose line was 24 Gy (range 20 to 28 Gy). In four patients, irradiation of two lesions in one treatment session was performed. Only one local failure was diagnosed during follow-up and occurred 1.4 months after treatment. All other lesions were locally controlled during a mean follow-up period of 5.4 months (range 1.4 to 20.2 months). All patients have deceased during follow-up and mOS was 8.3 months (range 4.2 to 31.9 months). Of these patients, 71.4% survived the first 6 months after RT and after 12 months, 2 patients were still alive (18%).

## Discussion

This is the first report describing a rare group of patients with metastasized PAC in bone, liver and brain undergoing palliative RT treatment. According to our analysis, mOS of the entire group is comparably short at 4.2 months after initiation of RT. Palliation or prevention of clinically relevant symptoms was achieved in a high number of patients although follow-up with medical imaging and clinical examination was very short; due to the palliative nature of the treatment, in some patients follow-up did not included imaging or was only performed depending on the performance status of the patient. In our subset of patients with bone metastasis, symptomatic response was recorded in 85% after RT and proved therefore a trend in efficacy. Local failure after RS treatment of liver metastasis in oligometastasized patients was only recorded in one lesion out of eleven lesions in seven patients. RS is a safe and a non-invasive treatment method in this patient group and can be applied in one session without the disadvantages of prolonged treatment duration over several weeks.

A relatively short follow-up period may be explained by the generally dismal prognosis of this patient group with reported median survival times of only several months or even weeks [6,7]. Even though efficacy of RT in palliating symptoms derived from brain or bone metastasis is well known, treatment duration plays an important role especially in poor-prognosis patients. The radiation oncologist has to decide carefully about the appropriate treatment schedule in terms of dose prescription and duration. Customizing radiation treatment in cancer patients with advanced and metastatic disease with respect to their remaining lifetime is difficult and in most cases depends on the physicians’ personal opinion. Gripp *et al*. examined how palliative patients’ survival related to physicians’ subjective prediction and objective prognostic factors and concluded that prediction was poor, especially in patients with a remaining life expectancy of less than one month [17]. In general, clinician-predicted survival (CPS) is controversial and many reports point to imprecise and overly optimistic predictions in terminally ill cancer patients [25-29].

Recently Holmebakk *et al*. described CPS by surgeons for palliative patients and found a prognostic accuracy of only 28% for the subgroup of PAC patients [30]. Clinical researchers from the MSKCC therefore invented a normogram for the prediction of an individual PAC patient that was recently validated from de Castro *et al*. [31].

In a subgroup of hepatocellular and cholangiocellular carcinoma patients with bone metastasis, our group recently found a comparable poor mOS with 3.4 months compared to 3.1 months of the corresponding subgroup in our current analysis [12]. Analysis of prescribed RT protocols demonstrated a clear trend towards longer treatment schedules and thus longer hospitalization rates in terminally ill cancer patients. In a large number of cases, RT protocol included 20 fractions of 2 Gy and single fraction treatments were not recorded. A total of 30% of all treatments (20/66 lesions), patients were treated for more than 20% of their remaining lifetime, and in five patients treatment lasted longer than 50% of the remaining lifetime.

The dismal prognosis of our patient cohort is certainly based on the considerably high metastatic potential of the underlying PAC. All analyzed patients with brain metastases and two out of seven patients treated for liver metastases had further metastasis outside of these organs. A total of four patients of the liver metastasis group had multiple lesions. Therefore, PAC patients with metastatic disease are rather unlikely to present at an oligometastasized stage with one to four metastases. The poor prognosis of PAC patients is a result of a highly aggressive local and distant dissemination pattern of the disease. A group of pathologists from the Johns Hopkins University in Baltimore has recently performed an extensive autopsy study with PAC patients and found a local and distant disease progression in 30% and 70% respectively [32]. Beside the fact, that the group was able to correlate the DPC4-genetic status with a more metastatic subtype, they also quantified metastatic patterns in their report. In summary, the most frequent sites of metastatic lesions were the liver and the peritoneum in 80% and 48% of cases respectively.

In a large clinical subset of PAC patients with locally advanced, but initially not metastasized disease, that underwent neoadjuvant chemoradiation with gemcitabine in case of unresectable disease, our analysis showed newly diagnosed metastases during follow-up predominantly located in the liver (52%) and in the peritoneum (36%) [2]. This seems to be in accordance with the previously mentioned results from the autopsy study but with the limitation of a clinical point of view, and thus not detecting the subclinical disease and, therefore, having a diagnostic bias. However, main sites of metastatic disease are the peritoneum and the liver. Whereas the liver offers a putative target for RT, peritoneal disease dissemination requires systemic or even intra-peritoneal cytostatic therapy, for example, in clinical trials.

## Conclusion

However, according to our results we suggest to subdivide PAC patients in a metastasized stage of disease into a first group of patients with disseminated disease requiring more or less immediate palliative RT because of clinically relevant symptoms. The second group consists of metastasized PAC patients in an oligometastasized stage (one to four metastatic lesions) that may benefit from local ablative therapies such as fractionated RT or even RS, as did the patients undergoing RS and presenting an OS of more than twelve months. For the first group, one has to consider a rather short RT schedule in consideration of the bad prognosis and short OS to avoid unnecessary long RT protocols to the cost of the remaining lifetime. For the latter group, a RS procedure is an effective local ablative therapy and even in case of a rapidly progressive disease after treatment *alio loco*, the remaining lifetime will not be affected as much as by fractionated RT protocols, as previously mentioned.

## Abbreviations

PAC: pancreatic cancer; GEM: gemcitabine; OS: overall survival; mOS: median overall survival; RT: radiotherapy; LINAC: linear accelerator; CT: computed tomography; GTV: gross tumor volume; PTV: planning target volume; MRI: magnetic resonance imaging; CI: confidence interval; WBRT: whole brain radiotherapy; CNS: central nervous system; RS: radiosurgery; CPS: clinician- predicted survival; MSKCC: Memorial Sloan Kettering Cancer Center; DPC4: Deleted in pancreatic cancer locus 4.

## Competing interest

The authors declare that they have no competing interests.

## Authors’ contributions

DH, JD and SEC were responsible for patient treatment and care. ICB collected the patients’ data. DH collected the patients’ data, performed all statistical analyses and wrote the manuscript. DH, ICB, JD and SEC contributed to the analysis of data and revised the manuscript. SEC helped to write and finalized the manuscript. All authors helped with the interpretation of the data, read and approved the final manuscript.
